# Cytogenetic analysis of three sea catfish species (Teleostei, Siluriformes, Ariidae) with the first report of Ag-NOR in this fish family

**DOI:** 10.1590/S1415-47572010005000038

**Published:** 2010-06-01

**Authors:** Mauro Nirchio, Emanuel Ricardo Monteiro Martinez, Fausto Foresti, Claudio Oliveira

**Affiliations:** 1 Escuela de Ciencias Aplicadas del Mar, Universidad de Oriente, Isla de MargaritaVenezuela; 2 Departamento de Morfologia, Instituto de Biociências, Universidade Estadual Paulista Júlio de Mesquita Filho, Botucatu, SPBrazil

**Keywords:** cytogenetics, Ag-NORs, chromosome evolution, chromosome rearrangements, fish

## Abstract

Despite their ecological and economical importance, fishes of the family Ariidae are still genetically and cytogenetically poorly studied. Among the 133 known species of ariids, only eight have been karyotyped. Cytogenetic analyses performed on *Genidens barbus* and *Sciades herzbergii* revealed that both species have 2n = 56 chromosomes and *Cathorops* aff. *mapale* has 2n = 52 chromosomes: *Genidens barbus* has 10 Metacentrics (M), 14 Submetacentrics (SM), 26 Subtelocentrics (ST), and 6 Acrocentrics (A), *Sciades herzbergii* has 14M, 20SM, 18ST and 4A, whereas *Cathorops* aff. *mapale* has 14M, 20SM, and 18ST. The nucleolus organizer regions (NORs) were found in a single chromosome pair on the short arm of a large-sized ST pair in *Genidens barbus* and on the short arm of a middle-size SM pair in *Cathorops* aff. *mapale*. Multiple NORs on the short arms of two large-sized ST pairs were found in *Sciades herzbergii*. The occurrence of diploid numbers ranging from 2n = 52 through 56 chromosomes and the presence of different karyotypic compositions, besides the number and position of NORs suggest that several numeric and structural chromosome rearrangements were fixed during the evolutionary history of this fish family.

The order Siluriformes (catfishes) has 3,088 species, divided into 36 families and 477 genera distributed worldwide, except for the coldest areas in the Southern and Northern hemispheres (Ferraris, 2007). There are only two predominantly marine families: Plotosidae and Ariidae. The family Ariidae, know as sea catfishes, includes 133 species distributed in 26 genera. It presents a worldwide distribution with species that live in marine and brackish water (Ferraris, 2007). Recent phylogenetic studies showed that the family Ariidae is monophyletic, but its relationships with other siluriforms remain unclear. Some authors suggest a close relationship with Pangasiidae, Ictaluridae, and mainly Anchariidae (de Pinna, 1998; Kailola, 2004; Hardman, 2005; Sullivan *et al.*, 2006; Betancur-R *et al.*, 2007).

Karyotypes of only eight ariid species have been described so far and showed diploid numbers ranging from 2n = 54 to 2n = 56 and complements mainly constituted of biarmed chromosomes (Table 1). Considering the worldwide distribution of Ariidae and its commercial importance, the main objective of the present study was to describe the karyotypes of *Cathorops* aff. *mapale*, *Genidens barbus,* and *Sciades herzbergii* for a better understanding of the karyotypic evolution and relationships among genera and species in the family.

The species studied were: *Genidens barbus* (3 males and 3 females) from Ubatuba, São Paulo, Brazil (23°26'00.8” S 45°01'01.7” W, LBP 2338), *Cathorops* aff. *mapale* (4 males) from Isla Margarita, Nova Esparta, Venezuela (10°57'39.6” S 64°10'26.4” W, LBP 6061), and *Sciades herzbergii* (8 unsexed specimens) from Isla Margarita, Nova Esparta, Venezuela (10°57'39.6” S 64°10'26.4” W, LBP 6060). Fishes were identified and deposited in the fish collection of the Laboratório de Biologia e Genética de Peixes (LBP), Departamento de Morfologia, Instituto de Biociências, Universidade Estadual Paulista, São Paulo, Brazil and Escuela de Ciencias Aplicadas del Mar, Universidad de Oriente, Isla Margarita, Venezuela.

Mitotic chromosome preparations were performed according to the technique described by Foresti *et al.* (1993). Nucleolar organizer regions (Ag-NORs) were revealed by the silver-staining method (Howell and Black, 1980). The chromosome morphology was determined based on arm ratios, as proposed by Levan *et al.* (1964), and the chromosomes were classified according to their morphology as Metacentrics (M), Submetacentrics (SM), Subtelocentrics (ST), and Acrocentrics (A).

Cytogenetic analyses showed that *Genidens barbus* has 2n = 56 (10M+14SM+ 26ST+ 6A – Figure 1), *Cathorops* aff. *mapale* has 2n = 52 (14M+20SM+18ST – Figure 2a), and *Sciades herzbergii* has 2n = 56 (14M+20SM+18ST+4A *-* Figure 2b). Early karyotypic studies of *G. barbus* by Gomes *et al.* (1994, cited as *Netuma barba*) and *S. herzbergii* by Molina *et al.* (2004, cited as *Hexanematichthys herzbergii*) showed the same diploid numbers found in the present study. However, the karyotypic formulae previously described for these species were different from those found here (Table 1). These differences may be due to technical artifacts, such as differences in chromosome condensation, or may be real differences that should be checked in further studies covering the distribution area of these species.

The available data show that diploid numbers range from 2n = 52 to 2n = 56 among ariids and that the karyotypes are mainly constituted by biarmed chromosomes (Table 1). The 2n = 56 is the most common diploid number among ariids and occurrs in *Aspistor parkeri*, *Bagre bagre*, *Genidens barbus*, *G. genidens*, and *Sciades herzbergii* (Table 1). The diploid number 2n = 54 was reported for three species: *Ariopsis felis*, *Bagre marinus*, and *Cathorops* sp. (Table 1). The occurrence of 2n = 52 in *Cathorops* aff. *mapale* (present work) represents the lowest diploid number already described for ariids.

Up to now, only two species of *Cathorops* were cytogenetically investigated: *C.* aff. *mapale* (2n = 52 - present study) and one unidentified species, *Cathorops* sp., that has 2n = 54 (Gomes *et al.*, 1992). This difference in diploid numbers between species of a single genus was also found in *Bagre* (Fitzsimons *et al.*, 1988; Gomes *et al.*, 1990) (Table 1), suggesting that this is not a rare phenomenon among ariids. On the other hand, the two *Genidens* species analyzed (Gomes *et al.*, 1994) presented the same diploid number (Table 1).

The karyotypes of ariids are composed of all morphological types of chromosomes (Table 1). However, in some species such as those of the genus *Bagre* and *Sciades herzbergii*, a large number of metacentric and submetacentric chromosomes is observed, while in other species, such as *Bagre marinus*, a large number of subtelocentric and acrocentric chromosomes is observed (Table 1). This variation allows to hypothesize that many structural chromosome rearrangements were fixed during the karyotypic evolution among species of this family.

Oliveira and Gosztonyi (2000) studied the karyological evolution of the order Siluriformes, particularly of the family Diplomystidae, and reached the conclusion that the ancestral diploid number for this order is 2n = 56. Among the families closely related to Ariidae, species of Pangasiidae have about 2n = 60 chromosomes (Magtoon and Donsakul, 1987; Manosroi *et al.* 2003), and among Ictaluridae, the diploid numbers range from 2n = 40 to 72 (LeGrande and Cavender, 1980; LeGrande, 1981; Clark and Mathis, 1982; LeGrande *et al.*, 1984; Amemiya, 1986). This diploid number variation, as well as that observed among ariids, suggests that numeric chromosome rearrangements (fusions and fissions) may have had an important role in the karyotypic evolution of this group.

The present results on Ag-NORs location are the first described for ariids. The ariid species studied have single or multiple Ag-NORs (Table 1). The NORs were found on the short arm of a biggest-size ST pair in *Genidens barbus*, and on the short arm of a middle-size SM pair in *Cathorops* aff. *mapale*. Among the Ictaluridae species, only single NOR-bearing chromosomes have been observed (Amemiya *et al.*, 1986). This is also the most common condition in Siluriformes (Oliveira and Gosztonyi, 2000; Oliveira *et al.*, 2006) and even in Teleostei (Klinkhardt, 1998). On the other hand, multiple NORs on the short arm of two large-sized ST pairs were found in *Sciades herzbergii*, which reinforces the hypothesis that structural chromosome rearrangements were also fixed in the karyotypic evolution of ariids.

Chromosomal rearrangements, indicated by differences in diploid numbers and karyotypes among species and by morphological changes in the position of the Ag-NORs, are widespread among fishes of the family Ariidae (Table 1). These chromosome rearrangements may have played an important role in the karyotypic evolution of the family, as already suggested by LeGrande (1980) and Fitzsimons *et al.* (1988). However, additional data should be added to clarify the importance of these chromosomal rearrangements in the evolution of the species and genera of ariids.

**Figure 1:**
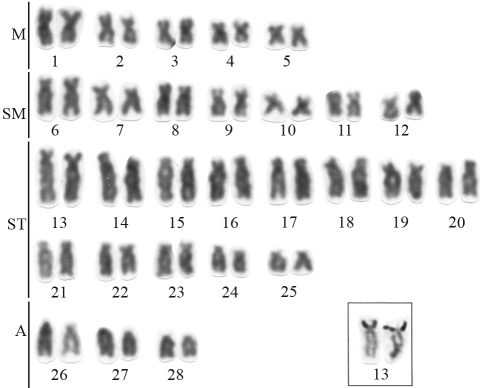
Giemsa stained karyotype of *Genidens barbus* with 2n = 56 chromosomes. In the inset, silver stained chromosomes showing the terminal Ag-NORs (black dots).

**Figure 2:**
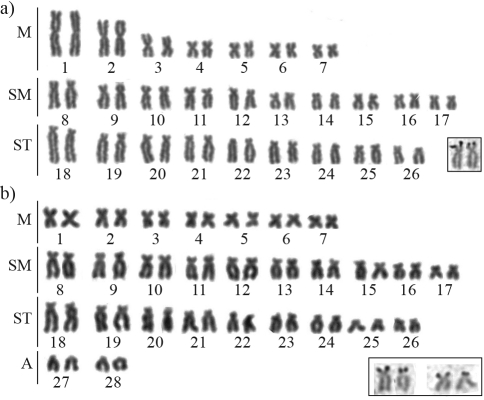
Giemsa stained karyotypes of (a) *Cathorops* aff. *mapale* with 2n = 52 chromosomes and (b) *Sciades herzbergii* with 2n = 56 chromosomes. In the inset, silver stained chromosomes showing terminal Ag-NORs (black dots).

## Figures and Tables

**Table 1:** Cytogenetic data available for the family Ariidae. Names in parentheses are original citations corrected in the present study.

Species	Locality	2n	Karyotype	NORs	References
*Ariopsis felis* (= *Arius felis*)	Caminada Bay, Louisiana, USA	54	26M/SM+28ST/A	-	LeGrande (1980)
*Aspistor parkeri* (= *Arius parkeri*)	Cananéia Coast, São Paulo, Brazil	56	16m+16sm+22st+2t	-	Gomes *et al.* (1994)
*Bagre bagre*	Cananéia Coast, São Paulo, Brazil	56	24M+26SM+6ST	-	Gomes *et al.* (1990)
*Bagre marinus*	Northern Gulf of Mexico, Louisiana, USA	54	12m+8sm+34STT	-	Fitzsimons *et al.* (1988)
*Cathorops* aff. *mapale*	Isla Margarita, Venezuela	52	14m+20sm+18st	1	Present study
*Cathorops sp.*	Cananéia Coast, São Paulo, Brazil	54	13m+13sm+28st	-	Gomes *et al.* (1992)
*Genidens barbus* (= *Netuma barba*)	Cananéia Coast, São Paulo, Brazil	56	18M+18SM+18st+2T	-	Gomes *et al.* (1994)
*Genidens barbus*	Ubatuba Coast, São Paulo, Brazil	56	10m+14sm+26st+6a	1	Present study
*Genidens genidens*	Cananéia Coast, São Paulo, Brazil	56	12m+20sm+20st+4t	-	Gomes *et al.* (1994)
*Sciades herzbergii* (= *Hexanematichthys herzbergii*)	Maracaibo Lake, Venezuela	56	24M+24SM+6st+2T	-	Molina *et al.* (2004)
*Sciades herzbergii*	Isla Margarita, Venezuela	56	14m+20sm+18st+4a	2	Present study

2n = diploid number; M = metacentrics; SM = submetacentrics; ST = subtelocentrics; A = acrocentrics; T = telocentrics; STT = subtelo-telocentrics; NORs = number of chromosome pairs with nucleolus organizer regions.
